# Phenotypic and Genomic Assessment of Antimicrobial Resistance and Virulence Factors Determinants in *Salmonella* Heidelberg Isolated from Broiler Chickens

**DOI:** 10.3390/ani15071003

**Published:** 2025-03-30

**Authors:** Arthur de Almeida Figueira, Thomas Salles Dias, Gisllany Alves Costa, Dayse Lima da Costa Abreu, Luciana dos Santos Medeiros, Virginia Léo de Almeida Pereira

**Affiliations:** 1 Postgraduate Program in Veterinary Medicine (Veterinary Hygiene and Processing Technology of Animal Products), Faculdade de Veterinária, Universidade Federal Fluminense, Niterói 24230-320, RJ, Brazil; arthurfigueira@id.uff.br (A.d.A.F.); gisllanyalves@id.uff.br (G.A.C.); dayseabreu@id.uff.br (D.L.d.C.A.); virginialeo@id.uff.br (V.L.d.A.P.); 2 Department of Preventive Veterinary Medicine, Faculdade de Veterinária, Universidade Federal Fluminense, Niteroi 24230-320, RJ, Brazil; luciana_medeiros@id.uff.br

**Keywords:** antimicrobial resistance, broiler chicken, foodborne infections, *Salmonella*, whole-genome sequencing

## Abstract

**Simple Summary:**

In our study, we investigated antimicrobial resistance in *Salmonella* Heidelberg, a bacterium commonly found in poultry and poultry food products. We analyzed 14 strains isolated from chickens in Brazil between 2013 and 2019, testing their phenotypic resistance to various antimicrobials and performing genomic analysis to identify the genes responsible for this resistance. Our results revealed a high frequency of resistance to commonly used antibiotics, such as cephalosporins, tetracyclines, and sulfonamides. All samples carried specific genetic mutations and resistance genes that could enhance their resistance and potentially increase their virulence. Notably, we identified genes typically associated with *Yersinia*, a pathogen that has been linked to more severe infections, suggesting that these *Salmonella* strains may pose a greater risk than previously expected. These findings underscore the need for continued surveillance to prevent foodborne illness and ensure the effectiveness of infection treatments.

**Abstract:**

*Salmonella* Heidelberg is frequently found in poultry and poultry products and is associated with antimicrobial resistance strains and infections and mortality in humans. Whole-genome sequencing is used to monitor and understand epidemiological factors related to antimicrobial resistance. This study aimed to characterize the phenotypic resistance and sequence the whole genome of *Salmonella* Heidelberg strains isolated from poultry products in Brazil. Fourteen *Salmonella* Heidelberg strains isolated from whole broiler chicken carcasses and portions in Brazil between 2013 and 2019 were used in this study. Genus confirmation was performed by polymerase chain reaction. The disk diffusion test was conducted to assess the phenotypical antimicrobial susceptibility of the strains. Whole-genome sequencing was carried out to investigate the presence of antimicrobial resistance genes, plasmids, multilocus sequence typing, and virulence-associated genes. A high frequency of phenotypic resistance to cephalosporins, tetracyclines, and sulfonamides was detected. All strains had mutations in *gyrA* and *parC* and contained the genes *tet(A)*, *fosA7*, and *sul*. The presence of genes originating from *Yersinia* pathogenicity islands was also detected. This study identified a high frequency of antimicrobial resistance in *Salmonella* Heidelberg strains from broilers slaughtered in different regions of Brazil, all belonging to the same sequence type (ST15) and associated with multiple resistance and virulence genes. The presence of the *Yersinia* high-pathogenicity island was detected, indicating potential virulence. These findings highlight the importance of continuously monitoring antimicrobial resistance to control and prevent foodborne infections and maintain the efficacy of treatments for human salmonellosis.

## 1. Introduction

*Salmonella enterica* can colonize animals and humans and is one of the most common foodborne microorganisms worldwide. The consumption of raw, undercooked, or contaminated chicken meat is considered to be one of the main sources of human infection [1]. There are more than 2400 known serotypes of *Salmonella*, and Heidelberg serovar is frequently associated with poultry and poultry products [2]. In Brazil, the world’s leading exporter of poultry meat, four serovars are controlled by the official surveillance program: Enteritidis, Typhimurium, Gallinarum and Pullorum [3]. Despite the lack of specific legislation focused on the *Heidelberg* serovar, in 2016, as a result of Normative Instruction 16, control and monitoring of *Salmonella* spp. were established in commercial poultry establishments for broiler chickens and turkeys, as well as in slaughterhouses for chickens, hens, turkeys, and breeders registered with the Federal Inspection Service [4]. Research on strains from the Brazilian poultry chain has detected a high prevalence of *Salmonella* Heidelberg, making it one of the serovars most commonly associated with poultry products [5,6,7]. The Heidelberg serovar has shown a significant increase in occurrence in both poultry and humans, and is often associated with severe infections. It can carry various virulence factors, including those found in the *Yersinia* high-pathogenicity island (HPI), which are associated with increased virulence and enhanced biofilm formation capacity, potentially facilitating its persistence in the environment [8]. Additionally, concern over antimicrobial resistance is particularly relevant, as multidrug-resistant strains have been found to be linked to outbreaks of salmonellosis in humans [9,10,11,12].

Antimicrobial resistance is a global threat to human and animal health and represents one of the greatest challenges of this generation. Antimicrobial resistance in *Salmonella* spp. requires continuous study, given the significance of this bacterial genus among foodborne pathogens [13]. Currently, the Centers for Disease Control and Prevention (CDC) and World Health Organization (WHO) consider non-typhoidal *Salmonella* strains that are resistant to fluoroquinolones and cephalosporins to be urgent threats to public health [14,15]. In Brazil, several studies have reported the emergence of *Salmonella* Heidelberg strains from poultry that have proven resistant to multiple drugs, including cephalosporins, fluoroquinolones, tetracyclines, and sulfonamides [5,16,17].

In response to the growing concerns about antimicrobial resistance in *Salmonella* Heidelberg strains that originate from poultry, advanced molecular tools have become critical when it comes to monitoring and understanding the spread of resistance. The use of whole-genome sequencing represents a significant advancement in microbial monitoring, providing definitive information on the relatedness of bacteria from different sources and their transfer of specific genes, including antimicrobial resistance genes [13]. Genomic surveillance of antimicrobial resistance is one of the strategies outlined by the WHO to mitigate the burden of infections caused by resistant microorganisms and guide the optimal use of existing antibiotics [18]. Therefore, we aimed to characterize the phenotypic antimicrobial resistance and perform whole-genome sequencing of *Salmonella* Heidelberg strains isolated from poultry products in Brazil in order to identify antimicrobial resistance genes and genes associated with virulence. This study noted a high frequency of antimicrobial resistance in *Salmonella* Heidelberg strains isolated from broiler chickens in Brazil, all belonging to sequence type ST15 and harboring multiple resistance and virulence genes, including those from *Yersinia* pathogenicity islands.

## 2. Materials and Methods

### 2.1. Bacterial Strains

**Figure 1:**
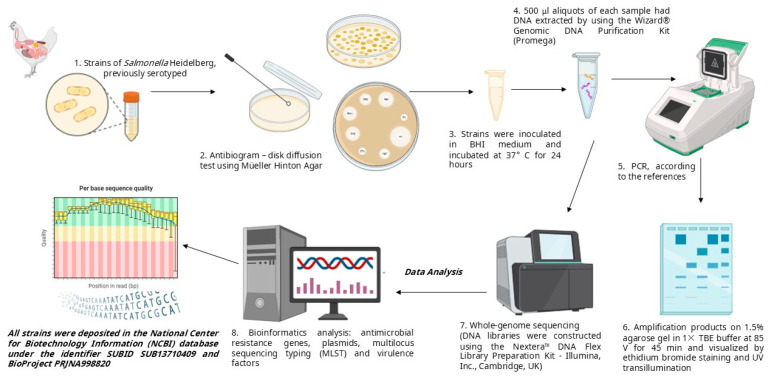
Representative scheme of methodology and experimental design of this study.

Fourteen strains of *Salmonella* Heidelberg originating from broiler carcasses and portions from the Central-West, Southeast, and South regions of Brazil were analyzed. These strains were stored at the Poultry Health Laboratory, Department of Veterinary Collective Health and Public Health, Faculty of Veterinary Medicine, Fluminense Federal University, between 2013 and 2019, and previously serotyped in the National Reference Center, Institute Oswaldo Cruz, Rio de Janeiro, Brazil (Figure 1).

### 2.2. Antibiogram

The disk diffusion test was performed for 19 antimicrobials using plates containing Müeller Hinton Agar (Merck^®^, Darmstadt, Germany), with the cut-off points defined by the Clinical and Laboratory Standards Institute [19,20]. The antimicrobial categories tested were aminoglycosides (Gentamicin-10 μg), carbapenems (Ertapenem-10 μg, Imipenem-10 μg, Meropenem-10 μg), 1st- and 2nd-generation cephalosporins (Cephalexin-30 μg, Cefalotin-30 μg, Cefoxitin-30 μg), 3rd-generation and 4th-generation cephalosporins (Cefotaxime-30 μg, Ceftazidime-30 μg, Ceftiofur-30 μg, Cefepime-30 μg), penicillins (Ampicillin-10 μg), penicillins + β-lactamase inhibitors (Amoxicillin + clavulanate-20/10 μg), monobactams (Aztreonam-30 μg), fluoroquinolones (Ciprofloxacin-5 μg, Enrofloxacin-5 μg), phenicols (Chloramphenicol-30 μg), tetracyclines (Tetracycline-30 μg), and sulfonamide (300 μg). The *Escherichia coli* strain ATCC 25922 was used as a control for the test.

### 2.3. DNA Extraction

The strains were inoculated in BHI medium and incubated at 37 °C for 24 h without agitation. After growth, DNA was extracted from 500 µL aliquots of each sample using the Wizard^®^ Genomic DNA Purification Kit (Promega, São Paulo, Brazil), according to the manufacturer’s recommendations. The DNA samples were evaluated for purity using a BioDrop Touch^®^ spectrophotometer (Biochrom Ltd., Cambridge, UK).

### 2.4. Polymerase Chain Reaction

To confirm the isolates at the genus level, we used the ST11 (AGC CAA CCA TTG CTA AAT TGG CGC A) and ST15 (GGT AGA AAT TCC CAG CGG GTA CTG) primer set, as described by Aabo et al. 1993 [21]. This primer set is highly specific to *Salmonella* species and produces an amplified fragment of 429 bp. For PCR analysis, the samples were required to have a minimum concentration of 20 ng/μL, with a 260/280 absorbance ratio ≅ 1.8 and a 260/230 ratio ≅ 2.0. The PCR reactions had a final volume of 25 μL containing 1X PCR buffer, 1.5 mM MgCl_2_, 5 μL of DNA, 0.2 μM of each primer and, 1U of Taq polymerase (Promega, São Paulo, Brazil), and the amplification was performed on a thermocycler model *T-100* (Bio-Rad Laboratories, Inc., Waltham, MA, USA). Samples were denatured at 94 °C for 2 min, and 35 cycles of amplification were performed at 95 °C for 30 s, 60 °C for 30 s, and 72 °C for 30 s. The reaction was completed by a final 10 min extension at 72 °C. *Salmonella* Enteritidis ATCC 13076 was used as a positive control and ultra-pure water was used as a negative control. Aliquots of amplification products were separated on 1.5% agarose gel in 1× TBE buffer at 85 V for 45 min and visualized by ethidium bromide staining and UV transillumination.

### 2.5. Whole-Genome Sequencing

The DNA libraries were constructed using the Nextera™ DNA Flex Library Preparation *Kit* (Illumina, Inc., Cambridge, UK). DNA fragments were sequenced using 150 bp paired-end libraries on the Illumina Miseq sequencer. The software FastQC (https://www.bioinformatics.babraham.ac.uk/projects/fastqc/, accessed on 1 June 2024) was used for quality control of the raw sequences for subsequent bioinformatics analyses. Genome assembly and annotation were performed by the Bacterial and Viral Bioinformatics Resource Center (https://www.bv-brc.org/, accessed on 24 June 2024). Bioinformatics analyses were carried out using online tools and databases available at the Center for Genomic Epidemiology (CGE) (www.genomicepidemiology.org) to investigate the presence of antimicrobial resistance genes (ResFinder 3.2), plasmids (PlasmidFinder 2.0), and for multilocus sequence typing (MLST 2.0). A search for virulence factors related to the *Yersinia* high-pathogenicity island was conducted using VFanalyzer in relation to the Virulence Factors Database (VFDB). All analyses were performed using standard parameters available on the platforms. The strains were deposited at the National Center for Biotechnology Information (NCBI) database under the identifier SUBID SUB13710409 and BioProject PRJNA998820 (Figure 1).

## 3. Results

Fourteen *Salmonella* Heidelberg strains, which were collected from various regions in Brazil between 2013 and 2019, were subjected to phenotypic antimicrobial resistance profiling and whole-genome sequencing. Table 1 details the percentages of antimicrobial resistance and Table 2 presents the sequence types, resistome, and phenotypic resistance profiles of the isolates. In the present study, a high frequency of phenotypic resistance to amoxicillin with clavulanic acid, ampicillin, cephalexin, cefalotin, ceftiofur, cefoxitin, ceftazidime, cefotaxime, tetracycline, and sulfonamide was detected in the *Salmonella* Heidelberg strains analyzed. None of the strains were resistant to the carbapenems tested. In relation to virulence, the genes *ybtT*, *irp2*, *psn/fyuA*, *ybtP*, *ybtQ*, *ybtU*, and *ybtX*, which were all associated with the HPI, were detected in 8 out of the 14 strains analyzed (57.14%).

## 4. Discussion

A high frequency of resistance to clinically important antimicrobials was observed in *Salmonella* Heidelberg strains isolated from cuts and carcasses of slaughtered chickens from different regions of Brazil. In recent years, *Salmonella* Heidelberg has emerged as the predominant serovar associated with the poultry chain in Brazil, exhibiting resistance to multiple classes of antimicrobials [17,22,23,24], and it has been identified as a common serovar associated with human salmonellosis in North and South America [9,25].

Similar to other studies, we detected high clonality among the *Salmonella* Heidelberg strains [6,7,26]. Studies that used the Pulsed-Field Gel Electrophoresis technique [6,7] reported difficulty in differentiating this serovar due to the identical banding patterns in strains from different origins. In the present study, all Heidelberg strains were classified as ST15, consistent with findings from other genomic studies involving strains isolated in Brazil [26,27]. Kipper et al. (2021) [26] reported that this single lineage of *Salmonella* Heidelberg serovar is widespread in the national poultry chain and is associated with multiple resistance and virulence genes. This may have occurred because serovars such as Enteritidis and Typhimurium are prioritized within the National Avian Health Program, established in 1994 [28]; therefore, the control of these serovars may have created an ecological niche that has allowed *Salmonella* Heidelberg to spread throughout the poultry chain. Possessing various genes related to antimicrobial resistance and survival under stressful conditions, common disinfection measures used in farm and slaughterhouse facilities may not be effective in eliminating this serovar. In a study by Voss-Rech and collaborators [29], *Salmonella* Heidelberg survived in recycled poultry litter, indicating that burning residual feathers and the bedding shaking procedure were not sufficient to interrupt the cycle of residual contamination.

The plasmids replicons detected in the genomes included IncX1, IncI, and IncC2, all of which have been previously described in other isolates of *Salmonella* Heidelberg [26,27,30]. These plasmids have incorporated various resistance genes, facilitating the spread of antimicrobial resistance in diverse environments [26,31]. Horizontal transfer of plasmids between different bacteria can lead to rapid dissemination of resistance, as co-selection due to the use of a single antimicrobial may select for resistance to multiple agents, making eradication of resistant strains more challenging [32]. Therefore, the presence of multiple plasmids with different replicons in *Salmonella* Heidelberg increases the complexity of controlling this microorganism [33,34].

Salmonellosis does not typically require antibiotic treatment, but in patients with immunosuppression, persistent diarrhea, or invasive infection, antibiotic therapy is necessary. Common first-line oral antibiotics for salmonellosis include fluoroquinolones and cephalosporins. Our findings agree with other studies [22,35] in which the main genetic element associated with cephalosporin resistance was the *bla*-CMY-2 gene. This gene is linked to resistance against penicillin, third-generation cephalosporins, β-lactamase inhibitors associated with β-lactams, and cephamycins. It has been identified as the primary determinant of resistance to ceftiofur [35], a cephalosporin used prophylactically in day-old chicks in some hatcheries around the world to mitigate early gastrointestinal infections caused by representatives of Enterobacteriaceae [36]. Touzain et al. (2018) [37] identified Avian Pathogenic *Escherichia coli* strains in diseased birds that carried the *bla*-CMY-2 gene on plasmids. This finding poses a serious risk to both animal welfare and public health, as the presence of *bla*-CMY-2 in poultry and poultry products could facilitate the transfer of these antibiotic-resistance genes to humans, potentially leading to ineffective treatments.

Dias et al., 2022 [16] reported a high frequency of non-susceptibility to ciprofloxacin in *Salmonella* Heidelberg using the minimum inhibitory concentration technique, with a low frequency of mobile elements associated with quinolone resistance. In the present study, all *Salmonella* Heidelberg strains possessed mutations in *gyr*A(S83F) and *par*C (T57S) associated with fluoroquinolone resistance, and no plasmid genes were detected in the analyzed strains. Single-nucleotide polymorphisms in the quinolone resistance-determining regions the *gyrA* and *parC* genes, which encode DNA gyrase and topoisomerase IV, respectively, can result in conformational changes in these enzymes. These changes prevent quinolones from binding to the DNA–enzyme complex, thereby maintaining the enzymes’ normal functions [38]. Despite all strains showing the main mutations correlated with phenotypic resistance to fluoroquinolones, 50% (7/14) of the strains exhibited phenotypical resistance to ciprofloxacin or enrofloxacin.

The genes *aac(6′)-Iaa*, *tet(A)*, *sul2*, and *fosA7* were present in all analyzed genomes, representing the main detected genes. The *aac(6′)-Iaa* gene, which is chromosomal and present in most *Salmonella* strains, encodes an enzyme that modifies aminoglycosides by acetylating drugs like tobramycin, kanamycin, and amikacin, but not gentamicin. According to the 2023 CLSI guidelines [19], even if these isolates appear susceptible to these antibiotics in vitro, they should not be reported as susceptible, as the strains may exhibit resistance in vivo. The *tet(A)* gene is linked to tetracycline resistance, *sul2* to sulfonamide resistance, and *fosA7* to fosfomycin resistance. These broad-spectrum antimicrobials have been widely used in animal production for many years due to their effectiveness and low cost. This prolonged use may have favored the selection of strains carrying these resistance genes within the production chain, facilitating their spread to food.

In relation to virulence, *Yersinia* HPI was detected in eight strains. This island was first reported in *Salmonella* Heidelberg from Brazil in 2021 [8]. The role of HPI is to facilitate the synthesis and transport of iron-chelating molecules in an environment that lacks this vital element. This process involves a set of genes organized in specific clusters aimed primarily at achieving iron binding in the surrounding environment. Once HPI binds to iron, it forms a molecular complex recognized by specific sites on the surface of the host cell [39]. *Yersinia* HPI in *Salmonella* can enhance virulence, posing a significant public health concern [8], may affect the regulatory protein of the HPI, and may have broader regulatory effects, influencing the expression of virulence factors or other genes beyond those associated with the island, enhancing the organism’s ability to initiate infection. Although *Salmonella* possesses several iron-acquisition systems, yersiniabactin’s high affinity for Fe^3+^ and its potential to suppress the host immune response may provide a significant advantage. The HPI could improve the organism environmental fitness and persistence, further aiding its survival [40].

A high degree of clonality was observed among *Salmonella* Heidelberg isolates from various regions and years in Brazil in this study, consistent with previous reports [6,26]. Kipper et al., 2021 [26] identified a multidrug-resistant *Salmonella* Heidelberg lineage circulating within Brazilian commercial poultry flocks, offering critical insights into the recent introduction of this lineage in 2004 and its subsequent population expansion. The rapid dissemination during the initial years is likely attributable to introduction via the poultry production pyramid, particularly through breeder flocks and hatcheries. In the following years, both horizontal and vertical transmission likely played a role in *Salmonella* Heidelberg contamination of broilers during pre-harvest and further along the production chain [26].

## 5. Conclusions

This study identified a high frequency of phenotypic resistance to medically important antimicrobials in *Salmonella* Heidelberg strains isolated from slaughtered chickens in different regions of Brazil. Genomic analyses revealed high clonality among the strains, all belonging to the same clone (ST15), which was associated with multiple resistance and virulence genes. The presence of the *Yersinia* HPI was also detected, indicating the potential virulence of the studied strains. Given these findings, it is crucial to implement targeted public health interventions. Future research should focus on developing and evaluating effective strategies to control antimicrobial resistance in poultry. Additionally, improving hygiene practices and biosecurity measures in poultry farms, along with stricter regulations in the broiler industry, could significantly reduce the risk of these resistant strains spreading. Enhanced surveillance and monitoring programs are also essential to detect and mitigate resistance trends, ensuring effective treatment options for human salmonellosis and reducing the risk of foodborne infection.

## Figures and Tables

**Table 1:** Percentage (%) of antimicrobial susceptibility of *Salmonella* Heidelberg strains isolated from broiler chicken carcasses in the disk diffusion test according to Clinical & Laboratory Standards Institute. R—resistant; I—intermediate resistance; S—susceptible.

Antimicrobial	R	I	S
Amoxicillin with clavulanic acid	71.43	7.14	21.43
Ampicillin	85.71	0.00	14.29
Cephalexin	78.57	0.00	21.43
Cefalotin	78.57	0.00	21.43
Cefoxitin	71.43	0.00	28.57
Ceftazidime	64.29	7.14	28.57
Cefotaxime	78.57	0.00	21.43
Ceftiofur *	57.14	21.43	21.43
Cefepime	7.14	28.57	64.29
Imipenem	0.00	0.00	100.00
Meropenem	0.00	0.00	100.00
Ertapenem	0.00	7.14	92.86
Aztreonam	57.14	7.14	35.71
Gentamicin	7.14	0.00	92.86
Ciprofloxacin	7.14	42.86	50.00
Enrofloxacin *	7.14	7.14	85.71
Chloramphenicol	21.43	0.00	78.57
Tetracycline	92.86	0.00	7.14
Sulfonamide	85.71	0.00	14.29

* Criteria from CLSI VET 2020.

**Table 2:** Sequence type, resistome, and phenotypic resistance profile of *Salmonella* Heidelberg strains isolated from broiler chicken carcasses.

Strain	Region	Year	Source	ST	Resistance Genotype	Plasmids	Phenotypic Resistance Profile	Intermediate Resistance	HPI
Heidelberg	South	2013	Carcass	15	aac(6′)-Iaa, blaCMY-2, tet(A), sul2, gyrA:p.S83F, parC:p.T57S, fosA7	ColpVC, IncC, IncX1	AMC, AMP, CFE, CFL, CFO, CAZ, CTX, CTF, ATM, TET, SUL	CPM, CIP	Yes
Heidelberg	South	2013	Carcass	15	aac(6′)-Iaa, blaCMY-2, tet(A), sul2, gyrA:p.S83F, parC:p.T57S, fosA7	ColpVC, IncC, IncX1	AMC, AMP, CFE, CFL, CFO, CAZ, CTX, ATM, TET, SUL	CTF, CIP, ENO	No
Heidelberg	South	2013	Carcass	15	aac(6′)-Iaa, blaCMY-2, tet(A), sul2, gyrA:p.S83F, parC:p.T57S, fosA7	ColpVC, IncC, IncX1, IncI1-I(Alpha)	AMC, AMP, CFE, CFL, CFO, CAZ, CTX, CTF, TET, SUL	CPM, ATM, CIP	No
Heidelberg	Midwest	2016	Carcass	15	aac(6′)-Iaa, blaCMY-2, tet(A), sul2, gyrA:p.S83F, parC:p.T57S, fosA7	ColpVC, IncC, IncX1	AMP, CLO, TET	-	No
Heidelberg	South	2017	Carcass	15	aac(6′)-Iaa, gyrA (p.S83F), parC:p.T57S, sul2, tet(A), fosA7,	ColpVC, IncC, IncX1, IncI1-I(Alpha)	AMC, AMP, CFE, CFL, CFO, CAZ, CTX, CTF, ATM, ENO, TET, SUL	CPM, CIP, ENO	Yes
Heidelberg	South	2017	Carcass	15	aac(6′)-Iaa, gyrA (p.S83F), parC:p.T57S, sul2, tet(A), fosA7,	ColpVC, IncC, IncX1	AMC, AMP, CFE, CFL, CFO, CTX, CTF, ATM, TET, SUL	CAZ	Yes
Heidelberg	Southeast	2017	Carcass	15	aac(6′)-Iaa, gyrA (p.S83F), parC:p.T57S, sul2, tet(A), fosA7,	ColpVC, IncC	AMC, AMP, CFE, CFL, CFO, CAZ, CTX, CTF, ATM, TET, SUL	-	No
Heidelberg	Southeast	2017	Carcass	15	aac(6′)-Iaa, gyrA (p.S83F), parC:p.T57S sul2, tet(A), fosA7	ColpVC, IncC, IncX1, IncI1-I(Alpha)	AMC, AMP, CFE, CFL, CFO, CAZ, CTX, CTF, ATM, TET, SUL	-	No
Heidelberg	Southeast	2017	Carcass	15	aac(6′)-Iaa, gyrA (p.S83F), parC:p.T57S sul2, tet(A), fosA7	ColpVC, IncC	AMC, AMP, CFE, CFL, CFO, CAZ, CTX, CTF, ATM, TET, SUL	CPM, ERT, CIP	Yes
Heidelberg	Southeast	2017	Carcass	15	aac(6′)-Iaa, blaCMY-2, tet(A), sul2, gyrA:p.S83F, parC:p.T57S, fosA7	ColpVC, IncC, IncX1, IncI1-I(Alpha)	AMC, AMP, CFE, CFL, CFO, CAZ, CTX, CTF, ATM, TET, SUL	-	Yes
Heidelberg	Southeast	2017	Carcass	15	aac(6′)-Iaa, gyrA (p.S83F), parC:p.T57S sul2, tet(A), fosA7	ColpVC, IncC	AMC, AMP, CFE, CFL, CFO, CAZ, CTX, ATM, TET, SUL	CTF, CIP, ENO	Yes
Heidelberg	South	2019	Carcass	15	aac(6′)-Iaa, blaCMY-2, tet(A), sul2, gyrA:p.S83F, parC:p.T57S, fosA7	ColpVC, IncC, IncX1, IncI1-I(Alpha)	SUL	-	No
Heidelberg	Southeast	2019	Retail	15	aac(6′)-Iaa, blaCMY-2, tet(A), sul2, gyrA:p.S83F, parC:p.T57S, fosA7	ColpVC, IncC, IncX1, IncI1-I(Alpha)	AMP, CFE, CFL, CTX, CTF, CPM, ATM, GEN, CLO, SUL	AMC, CIP	Yes
Heidelberg	South	2019	Carcass	15	aac(6′)-Iaa, blaCMY-2, tet(A), sul2, gyrA:p.S83F, parC:p.T57S, fosA7	ColpVC, IncC, IncX1, IncI1-I(Alpha)	CIP, CLO	-	Yes

ST: Sequence type. AMC = amoxicillin + clavulanic acid, AMP = ampicillin, CFE = cephalexin, CFL = cefalotin, CFO = cefoxitin, CAZ = ceftazidime, CTX = cefotaxime, CTF = ceftiofur, CPM = cefepime, IPM = imipenem, MPM = meropenem, ERT = ertapenem; ATM = aztreonam, CIP = ciprofloxacin, ENO = enrofloxacin, GEN = gentamicin, CLO = chloramphenicol, TET = tetracycline, SUL = sulfonamide, HPI = *Yersinia* high-pathogenicity island.

## Data Availability

The data presented in this study are available upon reasonable request from the authors.
